# Contrasting Individual-Specific Resilience and Compensation Personalization Frameworks: The Case of Rumination

**DOI:** 10.1016/j.bpsgos.2025.100478

**Published:** 2025-03-03

**Authors:** Sigal Zilcha-Mano

**Affiliations:** School of Psychological Science, University of Haifa, Haifa, Israel

**Keywords:** Capitalization, Compensation, Complementing, Mechanism of change, Personalized treatment, Resilience, Rumination

## Abstract

**Background:**

Rumination has been identified as a potential mechanism of therapeutic change, particularly in directive and focused psychotherapies for depression. Previous research has predominantly focused on either trait-like individual differences or state-like changes in rumination without integrating these aspects. In the current study, we propose a computational approach to investigating whether rumination serves as a compensatory or a resilience mechanism by integrating trait-like and state-like effects.

**Methods:**

Rumination and depressive symptoms were assessed (in *N* = 100) pretreatment and repeatedly throughout treatment. Mixed-level models were used to examine whether pretreatment trait-like rumination interacted with a time-variant variable of in-treatment state-like changes in rumination to predict subsequent changes in treatment outcomes. These models were used to determine whether individuals with higher or lower pretreatment trait-like levels of rumination benefited more from state-like reductions in rumination, thus contrasting the compensatory and resilience theoretical frameworks.

**Results:**

As hypothesized, the findings supported the compensatory framework; individuals with higher pretreatment trait-like levels of rumination benefited most from greater state-like reductions in rumination during treatment, as evidenced by greater subsequent symptom reduction (*p* = .04).

**Conclusions:**

The findings refine our understanding of rumination as an individual-specific mechanism of therapeutic change, dependent on an individual’s trait-like levels of rumination. The proposed computational approach enabled an empirical comparison of the 2 main theoretical frameworks of treatment personalization, compensatory and resilience, offering new insights into mechanisms that drive therapeutic change. Future studies could leverage the paradigm proposed here to examine for which patients and in what contexts mechanisms of change function as compensatory versus resilience mechanisms.

## Plain Language Summary

In this study, the authors investigated how rumination, a repetitive pattern of negative thinking, affects therapy outcomes for depression. Two competing theoretical frameworks were explored to understand how rumination functions during treatment, as a compensatory mechanism or as a resilience mechanism. A compensatory mechanism suggests that individuals with high levels of rumination before treatment benefit most from reducing it during therapy. In contrast, a resilience mechanism suggests that individuals with inherently lower rumination levels gain additional benefits by further reducing it during treatment.

The findings are consistent with the compensatory framework, showing that individuals with higher pretreatment rumination experienced the greatest symptom reduction from decreases in rumination during therapy. In contrast, individuals with lower pretreatment rumination levels showed less improvement from decreases in rumination during therapy, suggesting that the resilience framework is less applicable.

This study emphasizes the importance of tailoring therapy to individual needs and focusing on reducing high rumination levels to optimize treatment outcomes. The findings provide a framework for personalizing therapy based on whether rumination is a risk factor to be mitigated or a resource to be harnessed, thereby paving the way for more effective, personalized treatment strategies.

Rumination is characterized by repetitive and negative self-focused thoughts concerning various aspects of one’s identity and life (1). These thoughts are typically abstract, conscious, evaluative, and self-referential, often centering on the causes, meaning, and repercussions of distressing experiences (2,3). The content of ruminative thinking commonly revolves around personal relationships and interpersonal interactions and is triggered by social situations (4,5). Rumination has been identified as an important factor in the onset and maintenance of depression (6). Among individuals with major depressive disorder (MDD), rumination has been associated with increased symptom severity, poorer treatment response, higher relapse risk, and increased suicide risk (7,8). For example, rumination has been found to predict the onset of a subsequent major depressive episode in individuals without depression (9, 10, 11) as well as in individuals who were previously depressed (12). Consequently, it has been suggested that it is a crucial mechanism of change in the treatment of depression (13,14).

Given its potential importance as a mechanism of change, studies have increasingly examined whether treatment for depression leads to improvements in rumination and, if so, whether such improvement is associated with better treatment outcomes, as manifested in greater reductions in depressive symptoms. A meta-analysis by Spinhoven *et al.* (14), which included 36 studies (*N* = 3307 individuals), suggested that a wide range of active treatments for depression (e.g., cognitive behavioral therapy, mindfulness-based cognitive therapy, cognitive training, antidepressant medication, counseling) reduce repetitive negative thinking, particularly rumination, compared with control conditions. Further analyses indicated that more directive and focused treatments resulted in greater reductions in rumination. Additionally, lower levels of repetitive negative thinking, especially rumination, posttreatment were associated with lower levels of depression posttreatment, with the effect being particularly pronounced in more directive and focused treatments. However, there was notable heterogeneity in the effects, which was not entirely explained by the type of treatment. Understanding this heterogeneity is important for identifying individuals for whom improvements in rumination result in better treatment outcomes, thus paving the way for personalized treatment based on rumination (15).

When seeking to personalize treatment based on a critical mechanism, 2 competing theoretical frameworks of treatment personalization are especially instrumental: Rumination can serve as a compensation (16,17) and/or a resilience/capitalization mechanism (18, 19, 20). These 2 theoretical frameworks for treatment personalization rely on differentiating between 2 distinct components of any mechanism of change (19,21): trait-like levels of the mechanism, which differentiate individuals prior to treatment, and state-like changes in the mechanism, which occur within individuals as a result of targeting the mechanism during treatment (22). In the compensation framework, individuals with higher pretreatment trait-like levels of rumination benefit more from a state-like reduction in rumination occurring throughout treatment than individuals with lower trait-like levels of rumination. This suggests that individuals gain the most from compensating for their poorer maladaptive trait-like tendency to ruminate. In contrast, the resilience/capitalization framework posits that individuals with lower (vs. higher) pretreatment trait-like levels of rumination benefit most from a state-like reduction in rumination occurring during treatment, so that individuals with inherently lower levels of rumination can capitalize on this trait to achieve further reductions during treatment. In other words, whereas the former approach views rumination as a risk factor to be mitigated in treatment, the latter considers it to be a resilience factor to capitalize on.

Based on evolutionary theoretical conceptualizations, it can be speculated that rumination serves as a compensatory mechanism because of its potential beneficial function when kept at adaptive levels. Rumination is seen as having evolved to force individuals to deeply contemplate solutions to complex social problems that might otherwise lead to exclusion from the group. In our ancestral environment, being separated from the social group meant almost certain death, because a solitary primate would either starve or fall prey to predators (13,23). Therefore, given the potential benefits of adaptive levels of rumination, individuals with already relatively low trait-like levels of rumination (efficient/constructive rumination) are not expected to benefit from further reductions. This makes the resilience model of rumination as a mechanism of change less likely. In contrast, high trait-like levels of rumination are not adaptive at the individual level (inefficient/unconstructive rumination), which suggests that individuals with high trait-like levels of rumination benefit most from state-like reductions in rumination, supporting a compensatory role of rumination as a mechanism.

The overarching goal of the current study was to test whether rumination serves as a compensatory mechanism, whereby individuals with higher pretreatment levels of rumination benefit more from state-like reductions that occur throughout treatment (hypothesis 1). Given the accumulating evidence on the greater role of rumination as a mechanism of change in more directive and focused treatments, we hypothesized that the compensatory effect of rumination would be more pronounced in such treatments. In this study, we contrasted 2 types of treatments that differed in their level of directiveness and focus: a supportive-expressive treatment, which aims to improve maladaptive repetitive interpersonal patterns of thinking, behaving, and feeling; and a supportive nondirective condition. We hypothesized that the role of rumination as a compensatory mechanism would be more prominent in the supportive-expressive treatment than in the supportive condition (hypothesis 2). We used a computational approach to test these hypotheses. For the first hypothesis, we examined the cross-level interactive effect of rumination at the trait-like level (invariant, between-subject variance) and the state-like level (time-variant, within-subject variance) on subsequent changes in treatment outcomes. For the second hypothesis, we examined the cross-level interactive effect of treatment condition (supportive-expressive vs. supportive), trait-like rumination, and state-like rumination on subsequent changes in treatment outcomes.

## Methods and Materials

### Open Data, Open Materials, Open Code, and Preregistration

The analysis code appears online. The main analyses of the trial were preregistered before the first patient was recruited. The informed consent form signed by the participants stated that we would keep the data strictly confidential. The data are currently not available because to share them, we must obtain the consent of our participants and the Ethics Committee. We report on how we determined the sample size, all data exclusions, all manipulations, and all measures in the study protocol (24).

### Study Design and Participants

The sample included 100 individuals with MDD participating in the pilot or active phase of a randomized controlled trial (RCT) (25). Individuals participating in the RCT were randomized to one of the two 16-week short-term treatments, supportive-expressive treatment or supportive treatment, based on a minimization algorithm (26). Balancing factors were age (≥30 vs. <30), gender (male vs. female), family status (married/cohabiting vs. not married/cohabiting), baseline 17-item Hamilton Rating Scale for Depression (HRSD) score (27) (≥20 vs. <20), baseline attachment avoidance [≥3.5 vs. <3.5 on the avoidance subscale of Experience in Close Relationships [ECR] (28)], baseline attachment anxiety (≥3.5 vs. <3.5 on the anxiety subscale of the ECR), and personality disorders (present vs. absent). Assignment to treatment arm was conducted by an outside institution not involved in the study. Similar to many other RCTs for MDD and as hypothesized a priori, no differences in efficacy have been found between treatments. The trial protocol (24) and main outcome (25) provide additional details about the trial. All procedures were approved by the University of Haifa Institutional Review Board (protocol approval No. 186/15), and participants signed informed consent forms. The CONSORT (Consolidated Standards of Reporting Trials) diagram and therapist training details appear in the Supplement.

The inclusion and exclusion criteria specified below were used in patient recruitment. Inclusion criteria included the following: 1) met MDD diagnostic criteria assessed using structured clinical interviews for DSM-5, with scores above 14 on the 17-item HRSD (27) at 2 evaluations conducted 1 week apart, and current MDD based on the Mini-International Neuropsychiatric Interview (MINI) (29); 2) if on medication, patients’ dosage had been stable for at least 3 months before the start of the study, and patients were asked to maintain a stable dosage for the duration of treatment; 3) age between 18 and 60 years; 4) Hebrew language fluency; and 5) written informed consent. Exclusion criteria included the following: 1) current high risk of suicide or self-harm (HRSD suicide item >2); 2) current substance abuse disorder; 3) current or past schizophrenia, psychosis, bipolar disorder, or severe eating disorder requiring medical monitoring; 4) history of organic mental disease; and 5) currently in psychotherapy. Patients’ demographic and clinical characteristics are presented in Table S1.

### Treatments

Patients received sixteen 50-minute sessions of a time-limited therapy adapted for depression. They were randomized to receive either the supportive-expressive or the supportive treatment. The supportive-expressive treatment was designed to improve maladaptive, repetitive interpersonal patterns of thinking, behaving, and feeling that contribute to depressive symptoms. Within the supportive-expressive framework, rumination can be conceptualized as a nonproductive response of the self that perpetuates maladaptive interpersonal cycles, preventing individuals from fulfilling their interpersonal needs. By targeting these maladaptive self-responses, the treatment is aimed at fostering healthier, more fulfilling relationships, ultimately leading to a reduction in depressive symptoms (30,31). The supportive condition included the use of supportive techniques, such as affirmation and empathic validation. Unlike nonspecific supportive therapies, which have been found to be less effective than active treatments for depression (32), the supportive therapy used in this RCT was an active supportive treatment that has not been found to be less effective than other active therapies (25,33). For the supportive-expressive treatment, we used the Luborsky (34) manualized treatment. The supportive condition included all supportive techniques detailed in the manual used by Luborsky (34) but forbade the use of directive techniques (35).

### Measures

#### Psychiatric Disorders

The MINI (29) was administered to assess the presence and severity of depression and comorbid conditions.

#### Treatment Outcome

The outcome measure was the HRSD (27), a semistructured interview that contains 17 items assessing the patient’s symptoms in the preceding week. It was administered on a weekly basis. For the current study, the interrater reliability of the HRSD was intraclass correlation coefficient = 0.98.

#### Rumination

The Ruminative Response Scale (RRS) (2) was used to assess rumination severity, with the mean of the items serving as the final score. The internal reliability for the current study was 0.92. The pretreatment level of rumination at baseline served as the trait-like component. Rumination reports collected during treatment were made at weeks (W) 1, 2, 4, 8, 12, and 16. The change between 2 successive rumination reports collected during treatment served as the state-like components (i.e., W2 − W1, W4 − W2, W8 − W4, W12 − W8), which resulted in 4 state-like rumination reports.

### Overview of Statistical Analyses

Analyses were conducted using a 3-level hierarchically nested model of observations across time, nested within patients, nested within therapists, using the proc mixed procedure in SAS. To test our hypotheses, we used the trait-like component of rumination, its state-like component, and their interaction as predictors of subsequent change in HRSD scores (model 1). Then, we tested whether the effect of the interaction between the trait-like and the state-like components on the outcome differed as a function of treatment condition. To do so, we introduced a 3-way interaction between treatment condition and the trait-like and state-like components of rumination, together with all the lower-order effects as predictors of subsequent change in HRSD scores (model 2).

The model equations for the 2-way interaction (model 1) and the 3-way interaction (model 2) were as follows:

(1) $$\;\text{Model}\;1.\;\Delta {\text{HRSD}}_{\text{ijt}+1}=\;b0\;+\;b1\;\times \;\text{Trait}-{\text{like}}_{i}\;+\;b2\;\times \;\text{State}-{\text{like}}_{\text{it}}\;+\;b3\;\times \;\text{Trait}-{\text{like}}_{i}\;\times \;\text{State}-{\text{like}}_{\text{it}}\;+\;e_{\_\text{ij}\left(t+1\right)}$$

(2) $$\;\text{Model}\;2.\;\Delta {\text{HRSD}}_{\text{ijt}+1}=\;b0\;+\;b1\;\times \;\text{Trait}-{\text{like}}_{i}\;+\;b2\;\times \;\text{State}-{\text{like}}_{\text{it}}\;+\;b3\;\times \;\text{Trait}-{\text{like}}_{i}\;\times \;\text{State}-{\text{like}}_{\text{it}}\;+\;b4\;\times \;\text{Trait}-{\text{like}}_{i}\;\times \;\text{State}-{\text{like}}_{\text{it}}\;\times \;{\text{TXCON}}_{i}\;+\;b5\;\times \;\text{Trait}-{\text{like}}_{i}\;\times \;{\text{TXCON}}_{i}\;+\;b6\;\times \;\text{State}-{\text{like}}_{\text{it}}\;\times \;{\text{TXCON}}_{i}\;+\;b7\;\times \;{\text{TXCON}}_{i}\;+\;e_{\_\text{ij}\left(t+1\right)}$$

where State-like_ijt_ and ΔHRSD_ijt_ are the state-like components of the mechanisms (referring to changes) and the change in HRSD scores of patient i treated by therapist j at session t, respectively; Trait-like_i_ and TXCON_i_ are the trait-like components of the mechanisms and treatment condition (supportive-expressive vs. supportive) of patient i, respectively; e__ijt_ is the random error, which has a normal distribution; and the covariance between e__ijt_ and e__uvs_ is σ_st_ if i = u and j = v, and 0 otherwise.

## Results

### Preliminary Analyses

Demographic and clinical characteristics of participants in the RCT, together with descriptive statistics for measures of rumination and depressive symptoms, are presented in Tables S1 and S2. The correlation between the trait-like and state-like components of rumination was found to be only moderate (*r* = −0.20), supporting their conceptual distinction. Additionally, both components showed low to moderate correlations with depressive symptoms. Specifically, correlations between trait-like rumination and changes in depressive symptoms during treatment ranged from 0.03 to 0.20, while correlations between state-like changes in rumination and changes in depressive symptoms, measured concurrently, ranged from 0.10 to 0.20.

### Model 1

**Figure 1:**
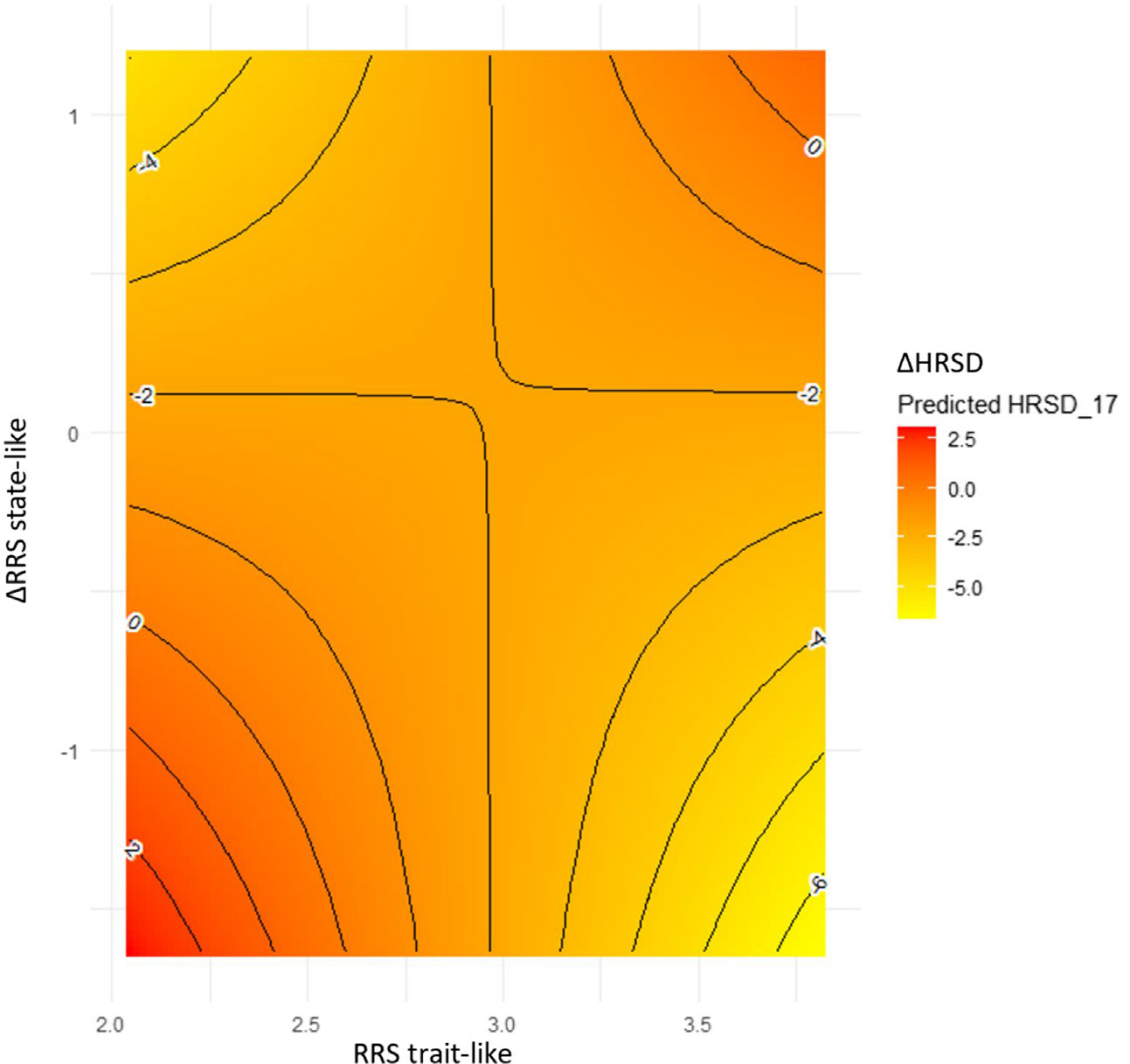
The interactive effect of trait-like and state-like rumination in predicting subsequent treatment outcomes. Ruminative Response Scale (RRS) trait-like indicates RRS at baseline, where higher levels mean more rumination; ΔRRS indicates state-like changes in rumination (session t + 1 − session t) so that more negative scores mean greater reduction as treatment progresses. ΔHRSD indicates change in depressive symptoms (session t + 1 − session t) so that more negative scores mean greater reduction as treatment progresses. For individuals with higher trait-like levels of rumination, greater (vs. less) state-like improvement in rumination resulted in greater reduction in symptoms. By contrast, for individuals with lower trait-like levels of rumination, greater (vs. less) state-like improvement in rumination resulted in less reduction in symptoms. HRSD, Hamilton Rating Scale for Depression.

The 2-way interaction was significant (Table 1). For individuals with higher trait-like levels of rumination (i.e., 1 SD above the mean), greater (vs. less) state-like improvement in rumination resulted in a greater reduction in symptoms. By contrast, for individuals with lower trait-like levels of rumination (i.e., 1 SD below the mean), greater (vs. less) state-like improvement in rumination resulted in less reduction in symptoms (Figure 1).

**Table 1:** The Interactive Effect of Trait-Like and State-Like Rumination in Predicting Subsequent Treatment Outcomes (Model 1)

Effect	Estimate	SE	*t* Value	Pr > |*t*|	Partial
Intercept	−1.06	1.16	*t*_214_ = −0.91	0.36	
Trait	−0.33	0.41	*t*_217_ = −0.81	0.42	0.001
State	−10.43	4.8	*t*_322_ = −2.16	0.03	0.011
Trait × State	3.39	1.66	*t*_317_ = 2.03	0.04	0.010

### Model 2

**Figure 2:**
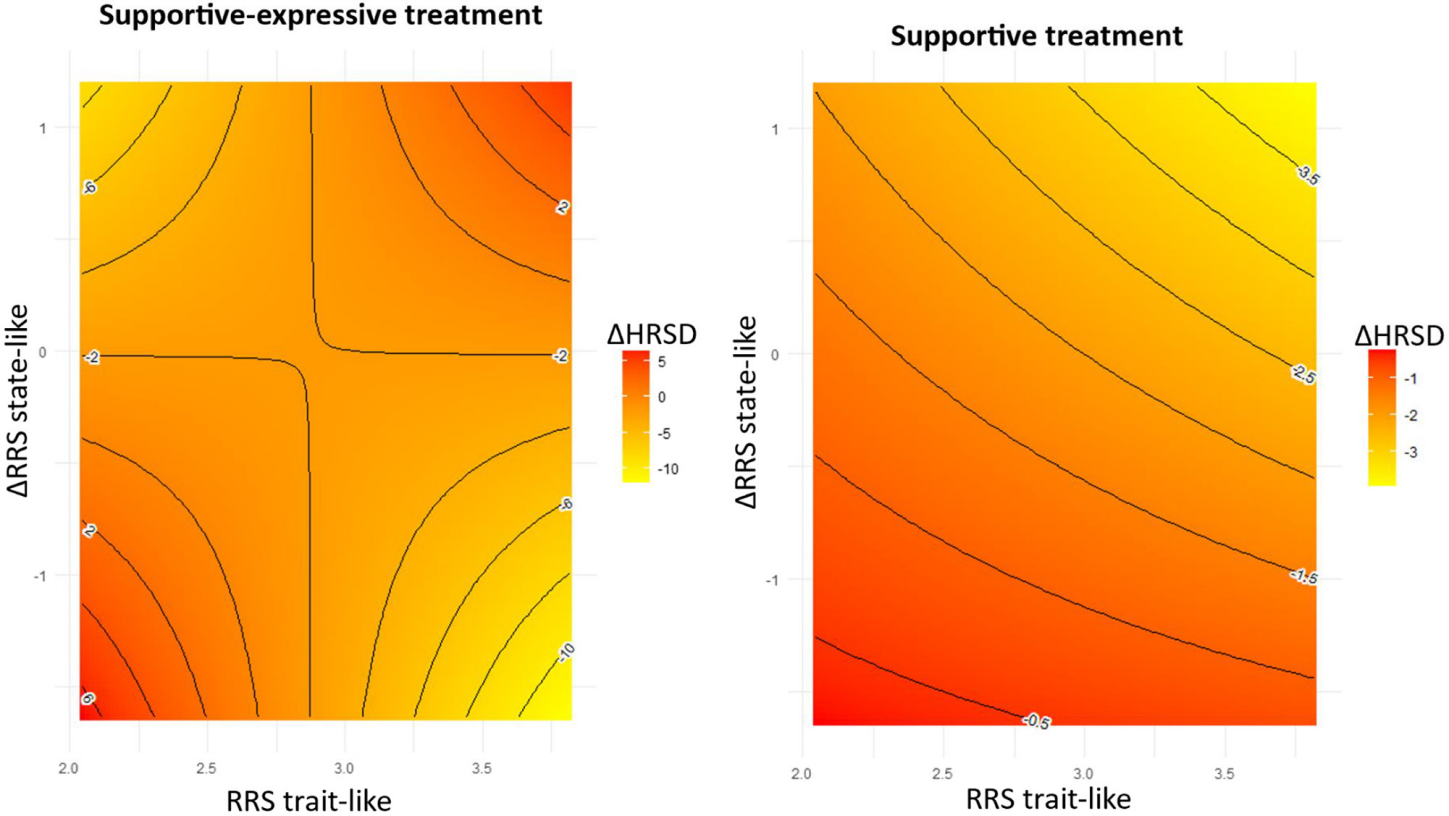
The moderating effect of treatment condition on the interactive effect of trait-like and state-like rumination in predicting subsequent treatment outcomes. Ruminative Response Scale (RRS) trait-like indicates RRS at baseline, where higher levels mean more rumination; ΔRRS indicates state-like change in rumination (session t + 1 − session t) so that more negative scores mean greater reduction as treatment progresses. ΔHRSD indicates change in depressive symptoms (session t + 1 − session t) so that more negative scores mean greater reduction as treatment progresses. HRSD, Hamilton Rating Scale for Depression.

The 3-way interaction was significant (Table 2). The 2-way interaction reported above was significant in supportive-expressive but not in supportive treatment (Figure 2).

**Table 2:** The Moderating Effect of Treatment Condition on the Interactive Effect of Trait-Like and State-Like Rumination in Predicting Subsequent Treatment Outcomes (Model 2)

Effect	Estimate	SE	*t* Value	Pr > |*t*|	Partial
Intercept	0.24	1.50	0.16	0.875	
TXCON (SET vs. ST)	−2.72	2.06	−1.32	0.189	0.004
Trait	−0.75	0.53	−1.41	0.163	0.002
Trait × TXCON (SET)	0.91	0.72	1.27	0.209	0.004
State	−0.72	6.48	−0.11	0.912	0.011
State × TXCON (SET)	−18.51	9.67	−1.91	0.057	0.009
Trait × State	−0.08	2.27	−0.04	0.971	0.010
Trait × State × TXCON	6.78	3.34	2.03	0.043	0.010

SET, supportive-expressive treatment; ST, supportive treatment; TXCON, treatment condition.

### Sensitivity Analysis

The same pattern of findings was observed after controlling for the effects of gender, socioeconomic status, age, pretreatment depressive symptom severity, and pretreatment anxiety symptom severity (see Tables S3 and S4). Exploratory post hoc analyses for each of the 3 subscales of the RRS appear in Tables S5–S10.

## Discussion

This study is the first to use a computational approach to examine whether rumination functions as an individual-specific compensatory or resilience/capitalizing mechanism, utilizing mixed-level moderators of both between-patient and within-patient effects. The findings are consistent with the hypothesis that rumination serves as a compensatory mechanism. Specifically, individuals with higher pretreatment trait-like levels of rumination experienced greater benefit from greater state-like reductions in rumination over the course of treatment, as manifested in greater subsequent symptom reduction. Conversely, individuals with lower pretreatment trait-like levels of rumination benefited least from greater state-like reductions in rumination over the course of treatment, as manifested in less symptom reduction. These results support the hypothesized compensatory role of rumination and provide no evidence for a resilience/capitalizing effect. As hypothesized, these effects were more pronounced in the supportive-expressive treatment, which focuses on addressing maladaptive repetitive interpersonal patterns of thinking, behaving, and feeling, than in the supportive nondirective condition.

In the current study, we investigated how trait-like rumination and treatment condition interact to moderate the within-patient effect of state-like rumination on subsequent changes in treatment outcomes. The classical approach of identifying moderators typically focuses on pretreatment between-subject characteristics of patients as a moderating factor of the effect of treatment condition on outcome (36,37). This classical approach answers the question of which treatment package is most effective for a given subpopulation of patients, as a function of their pretreatment values on the moderator. However, this approach refers to between-subject effects, whereas most of the common questions in clinical practice refer to within-subject effects (38,39). Moderators of within-subject effects show which mechanisms to target during a certain session to achieve subsequent improvement in treatment outcomes (38, 39, 40). The current study serves as a proof of concept in its computational approach, integrating moderators of between-subject and within-subject effects to answer the question of whether a given mechanism may serve as a compensatory or a resilience mechanism (19).

The computational approach proposed in this study integrates 2 traditionally separate lines of research on rumination: individual differences in trait-like levels of rumination and state-like changes in rumination. Previous studies have either focused on one aspect or conflated the two as the same entity. In research on trait-like differences, rumination is viewed as a relatively stable cognitive process, wherein individuals are characterized by a relatively stable tendency to ruminate about their feelings and problems (41,42). In contrast, research on state-like changes examined how experimental designs and therapeutic processes can induce changes in rumination. For example, emotional stressors have been shown to temporarily trigger state rumination in both observational and experimental settings (43, 44, 45). These 2 components of rumination also have distinct neural correlates. The trait-like tendency to ruminate in individuals with MDD has been associated with prefrontal hypoactivation (46, 47, 48), whereas state-like changes in rumination have been linked to dysregulated functional connectivity between subcortical limbic areas such as the amygdala and prefrontal cognitive control areas such as the ventrolateral prefrontal cortex (49,50). To our knowledge, no study has yet integrated both trait-like differences and state-like changes to investigate the role of rumination as a mechanism of change in treatment. This integration is made possible by the proposed computational approach, which allows the study of compensatory versus resilience mechanisms.

The current findings are consistent with the literature suggesting that rumination, in minimal doses, can serve an adaptive function (13). Rumination can aid in resolving problems or finding meaning, thus assuming a problem-solving and reflective form (51). However, when it occurs repeatedly in similar circumstances and is associated with low mood and unresolved goals, it can shift to a maladaptive and depressive focus, centered on negative thoughts and emotions (52). In such scenarios, rumination may become a habitual response to challenging life events, resulting in pathological consequences and diminished quality of life (53). Determining the right level and concrete nature of effective rumination should be the focus of future research, which should systematically investigate whether and how this adaptive level varies between individuals and across different life phases and circumstances within the same individual.

It should be noted that the use of trait-like and state-like components of rumination in this study differs slightly from their characterization in earlier, classical frameworks such as that of Nolen-Hoeksema (2). While trait-like rumination has been similarly described as a relatively stable characteristic of the individual, state-like rumination has traditionally been described in some literature [e.g., (54)] as a transient component triggered by situational factors (e.g., following an induced stressor). In contrast, in the current article, we have adopted the conceptualization of state-like changes in rumination as an integral part of the curative process of treatment and as the outcome of targeting rumination as a mechanism of therapeutic change. Once these state-like changes are consolidated into the individual’s characteristics, they have the potential to evolve into a new, relatively stable trait-like characteristic of the individual (19,21,22,55).

The computational approach proposed in this study should be tested on additional therapeutic mechanisms beyond rumination, such as worry. When studying rumination, it is important to apply this approach to diverse samples of patients and therapists to determine whether similar or varying results emerge, thereby adding valuable insights into its potential for personalized treatment. Replicating these findings using larger sample sizes is also essential. Although the number of repeated observations within individuals used in the current study allowed for detection of even small-to-medium-sized effects, the limited number of individuals restricts the power for between-subject effects. The findings regarding the moderating effect of the type of treatment attest to the importance of contrasting treatments that vary in their directives and focus, which is consistent with the accumulating literature on rumination (14). However, neither treatment focuses exclusively on targeting rumination. The supportive-expressive treatment is designed to address maladaptive repetitive interpersonal patterns (34). Although the content of ruminative thinking often revolves around personal and interpersonal relationships and is triggered by social situations (4,5), some ruminative thoughts may not be socially focused (48). Additionally, in this study, pretreatment values are considered relatively stable trait-like characteristics. Future research should explore the individual-specific dynamic nature of pretreatment trait-like levels of rumination measured on a day-to-day basis to provide a more comprehensive understanding (22).

While the current study serves as a proof of concept for the proposed framework’s potential to differentiate between compensatory and resilience mechanisms, it treats rumination as a unified construct. Future research could advance this framework by disentangling distinct elements of rumination, each of which may function independently as compensatory or resilience mechanisms. A critical distinction lies between problem-focused contemplation and abstract/meaning-focused contemplation or, alternatively, between concrete and abstract rumination (56). Problem-focused contemplation can be conceptualized as a resilience mechanism that promotes adaptive coping and problem resolution, whereas abstract/meaning-focused contemplation may function as a risk factor that requires targeted intervention to facilitate compensation during treatment. Similarly, the distinct subscales of the RRS, namely brooding, reflection, and depression-related rumination, offer a valuable lens to further deconstruct the construct of rumination (57). A post hoc analysis in the current study revealed potential differences among these subscales (see Tables S5–S10). These distinct elements of rumination may have different functions across individuals or within the same individual at different stages of life, depending on the specific challenges that they face. Understanding these nuanced patterns could enhance the precision of therapeutic interventions and deepen our understanding of rumination’s complex dynamics.

### Conclusions

The current study proposes a computational approach to contrast 2 key theoretical frameworks for treatment personalization, thereby complementing the growing body of literature that leverages computational methods for data-driven personalization approaches (15). Future research could benefit from integrating theory-driven and data-driven approaches in the same study. Moreover, it is important to clarify that the term individual-specific mechanism in this context refers to identifying which changes that occur during treatment are necessary for an individual with specific pretreatment characteristics to achieve subsequent improvement in outcomes rather than testing a mediation model. Future studies should combine the approach proposed here with a moderated-mediation framework conducted on large datasets to ensure sufficient power for testing these complex models. This integration will expand the understanding of personalized treatment mechanisms and improve the efficacy of therapeutic interventions.

The findings underscore the role of rumination as a compensatory rather than a resilience/capitalization mechanism. Specifically, individuals with higher pretreatment trait-like levels of rumination experienced the greatest symptom reduction from state-like reductions in rumination that occurred during treatment. The computational approach used in this study allows the comparison of the 2 primary theoretical frameworks of treatment personalization. It is grounded in a theoretical framework that emphasizes the crucial importance of disentangling pretreatment trait-like differences between individuals in mechanisms of change from state-like changes that occur within individuals during the course of treatment (19,55). It not only identifies who is most likely to benefit from targeting a specific mechanism but also identifies individuals for whom such targeting may lead to undesirable outcomes. This dual insight is crucial for optimizing personalized treatment strategies.

## Supplementary Material

Figure S1 and Tables S1–S10

Key Resources Table
