# Supplementary material for: Contrasting Individual-Specific Resilience and Compensation Personalization Frameworks: The Case of Rumination
Source: Biol Psychiatry Glob Open Sci. 2025 Mar 3;5(3):100478. doi: 10.1016/j.bpsgos.2025.100478 (PMC12008690; doi:10.1016/j.bpsgos.2025.100478)
Supplement: Figure S1 and Tables S1–S10 [file mmc1.pdf]

SUPPLEMENTARY INFORMATION

Contrasting Individual-Specific Resilience and Compensation Personalization Frameworks: The Case of Rumination

Zilcha-Mano

Table S1. Demographic and clinical characteristics of participants in the randomized controlled trial

Note. Values shown as % (n).

Variable	Supportive (n = 50)	Supportive-expressive (n = 50)	Total (N = 100)	Statistical test	p value
Demographics					
Age, years, M (SD)	31.02 (6.9)	31.5 (9.6)	31.2 (8.25)	t(98) =−.31	0.75
Education, years, M (SD)	14.5 (1.9)	13.9 (2.1)	14.2 (2)	t(98) = 1.26	0.21
Female	58 (29)	56 (28)	57 (57)	$\chi^2(1) = .41$	1
Income > average	24 (12)	26 (13)	25 (25)	$\chi^2(1) = .97$	.6
Married/cohabitating	16 (8)	16 (8)	16 (16)	$\chi^2(1) = 1.2$	1.2
Employed	76 (38)	60 (30)	68 (68)	$\chi^2(1) = 4.23$	0.050
Religion, Jewish	80 (40)	80 (40)	80 (80)	$\chi^2(1) = 0$	1
Clinical features					
Current medication, yes	14 (7)	14 (7)	14 (14)	$\chi^2(1) = 0$	1
Previous medication, yes	24 (12)	24 (12)	24 (24)	$\chi^2(1) = 0$	1
Previous psychotherapy, yes	52 (26)	40 (20)	46 (46)	$\chi^2(1) = 1.7$	0.23
Comorbidities					
Any disorder	72 (36)	70 (35)	71 (71)	$\chi^2(1) = .19$	.82
Any anxiety disorder	70 (35)	72 (36)	71 (71)	$\chi^2(1) = .05$	1
Any personality disorder	76 (38)	72 (36)	74 (74)	$\chi^2(1) = .2$	.82
Drop-outs	10 (5)	4 (2)	7 (7)	$\chi^2(1) = 1.38$	0.43
Remote treatment, yes	14 (7)	12 (6)	13 (13)	$\chi^2(1) = .08$	1

Note. Values shown as % (n).

Table S2. Mean and standard deviation (SD) of HRSD and RRS Scores at Each Time Point

Session	Mean	SD	Minimum	Maximum
**RRS**				
Baseline	2.85	0.44	2.05	3.82
Session 1	2.68	0.50	1.59	3.82
Session 2	2.65	0.51	1.41	3.77
Session 4	2.60	0.52	1.27	3.86
Session 8	2.53	0.62	1.09	3.86
Session 12	2.46	0.64	1.09	3.82
**HRSD**				
Baseline 1	20.96	3.70	14.00	30.00
Baseline 2	20.08	3.88	14.00	31.00
Session 1	18.02	4.93	6.00	28.00
Session 2	16.31	5.80	3.00	30.00
Session 4	14.23	5.90	3.00	27.00
Session 6	13.28	6.10	0.00	26.00
Session 8	11.85	6.37	0.00	28.00
Session 12	10.44	5.96	0.00	27.00
Session 16	7.95	5.79	0.00	24.00

Table S3. The interactive effect of trait-like and state-like rumination in predicting subsequent treatment outcomes (Model 1), controlling for demographic and clinical characteristics

Effect	Estimate	SE	t Value	Pr > |t|	partial
Intercept	-0.61	1.69	-0.36	0.72	
Trait	-0.38	0.48	-0.80	0.43	0.001
State	-11.35	4.78	-2.37	0.018	0.014
Trait*State	3.68	1.65	2.23	0.02	0.012
Age	0.021	0.021	1.02	0.31	0.003
Gender	0.34	0.35	0.95	0.34	0.002
Pre-treatment Anxiety	0.54	0.44	1.24	0.22	0.004
Pre-treatment Depression	-0.13	0.053	-2.39	0.018	0.014
Income > average	0.53	0.49	1.09	0.28	0.003

Table S4. The moderating effect of treatment condition on the interactive effect of trait-like and state-like rumination in predicting subsequent treatment outcomes (Model 2), controlling for demographic and clinical characteristics

Effect	Estimate	SE	t Value	Pr > |t|	partial
Intercept	-0.044	1.75	-0.03	0.98	
Txcond (SET vs ST)	-2.17	2.01	-1.08	0.28	0.003
Trait	-0.64	0.56	-1.13	0.26	0.001
Trait*Txcond (SET)	0.71	0.75	1.00	0.32	0.002
State	-1.31	6.42	-0.2	0.84	0.013
State*Txcond (SET)	-19.051	9.57	-1.99	0.049	0.009
Trait*State	0.049	2.25	0.02	0.98	0.012
Trait*State*Txcond	7.086	3.31	2.14	0.03	0.011
Age	0.026	0.02	1.43	0.16	0.005
Gender	0.29	0.30	0.97	0.33	0.002
Pre-treatment Anxiety	0.54	0.37	1.45	0.15	0.005
Pre-treatment Depression	-0.12	0.045	-2.66	0.009	0.017
Income > Average	0.612	0.42	1.45	0.15	0.005

Table S5. The interactive effect of trait-like and state-like reflective rumination, a subscale of the RRS, in predicting subsequent treatment outcomes (Model 1)

Effect	Estimate	SE	t Value	Pr > |t|	partial
Intercept	-1.67	0.79	-2.11	0.036	
Trait	-0.022	0.06	-0.37	0.71	0.0003
State	-1.41	0.52	-2.70	0.007	0.018
Trait*State	0.11	0.04	2.74	0.006	0.018

Table S6. The moderating effect of treatment condition on the interactive effect of trait-like and state-like reflective rumination, a subscale of the RRS, in predicting subsequent treatment outcomes (Model 2)

Effect	Estimate	SE	t Value	Pr > |t|	partial
Intercept	-0.33	1.01	-0.32	0.75	
Txcond (SET vs ST)	-2.48	1.38	-1.80	0.07	0.008
Trait	-0.12	0.077	-1.54	0.12	0.0006
Trait*Txcond (SET)	0.18	0.11	1.73	0.084	0.007
State	-0.58	0.76	-0.76	0.45	0.015
State*Txcond (SET)	-1.58	1.09	-1.44	0.15	0.005
Trait*State	0.035	0.061	0.57	0.57	0.015
Trait*State*Txcond	0.14	0.084	1.63	0.10	0.007

Table S7. The interactive effect of trait-like and state-like depression-related rumination, a subscale of the RRS, in predicting subsequent treatment outcomes (Model 1)

Effect	Estimate	SE	t Value	Pr > |t|	Partial
Intercept	-0.57	1.12	-0.51	0.61	
Trait	-0.042	0.031	-1.32	0.19	0.004
State	-0.52	0.32	-1.62	0.11	0.006
Trait*State	0.012	0.009	1.42	0.15	0.005

Table S8. The moderating effect of treatment condition on the interactive effect of trait-like and state-like depression-related rumination, a subscale of the RRS, in predicting subsequent treatment outcomes (Model 2)

Effect	Estimate	SE	t Value	Pr > |t|	partial
Intercept	-0.38	1.32	-0.29	0.77	
Txcond (SET vs ST)	-0.79	1.94	-0.41	0.68	0.0004
Trait	-0.043	0.038	-1.13	0.26	0.004
Trait*Txcond (SET)	0.016	0.055	0.29	0.77	0.0002
State	-0.031	0.44	-0.07	0.94	0.007
State*Txcond (SET)	-1.01	0.65	-1.56	0.12	0.006
Trait*State	-0.0004	0.012	-0.03	0.97	0.006
Trait*State*Txcond	0.028	0.018	1.56	0.12	0.006

Table S9. The interactive effect of trait-like and state-like brooding rumination, a subscale of the RRS, in predicting subsequent treatment outcomes (Model 1)

Effect	Estimate	SE	t Value	Pr > |t|	Partial
Intercept	-1.74	0.86	-2.03	0.044	
Trait	-0.017	0.057	-0.30	0.76	0.0002
State	-0.95	0.53	-1.77	0.077	0.008
Trait*State	0.06	0.036	1.68	0.09	0.007

Table S10. The moderating effect of treatment condition on the interactive effect of trait-like and state-like brooding rumination, a subscale of the RRS, in predicting subsequent treatment outcomes (Model 2)

Effect	Estimate	SE	t Value	Pr > |t|	partial
Intercept	-1.34	1.02	-1.31	0.19	
Txcond (SET vs ST)	-0.79	1.49	-0.53	0.59	0.0007
Trait	-0.034	0.07	-0.50	0.62	0.0002
Trait*Txcond (SET)	0.039	0.09	0.39	0.69	0.0004
State	-0.38	0.76	-0.50	0.62	0.005
State*Txcond (SET)	-0.86	1.10	-0.78	0.43	0.001
Trait*State	0.017	0.053	0.32	0.75	0.005
Trait*State*Txcond	0.07	0.074	0.94	0.35	0.002

Figure S1. Flow of participants in the study comparing Supportive Treatment vs. Supportive-Expressive Treatment for major depressive disorder

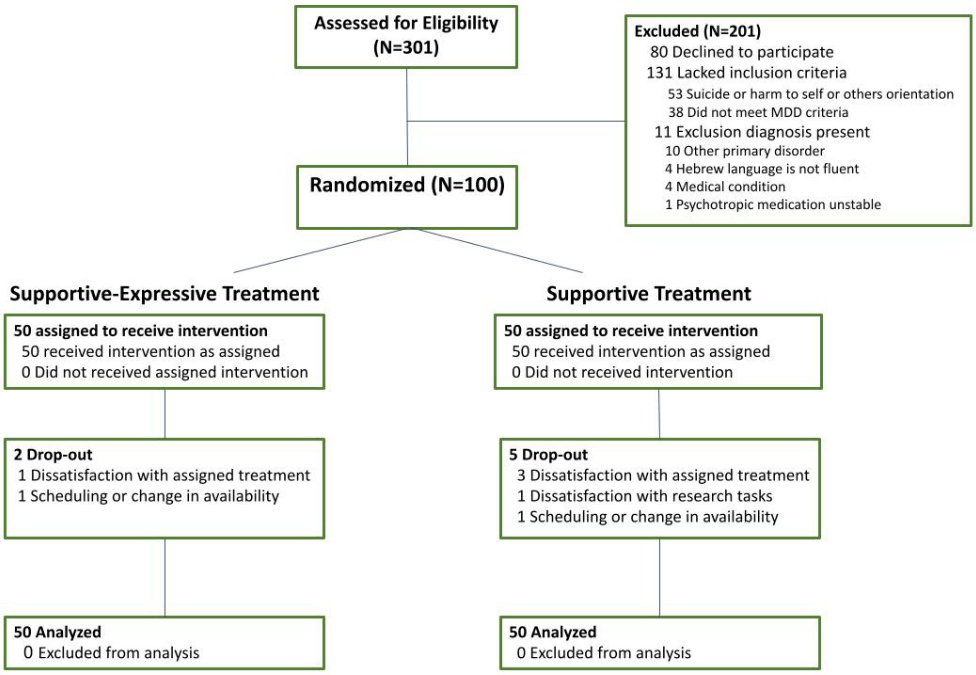

Therapists

Therapists acted as their own controls, providing treatment in both conditions. Eight therapists with at least five years of expertise in psychodynamic treatment attended a 20-hour training workshop in supportive and expressive techniques. Therapists completed treatment of two pilot cases, one of each treatment condition and demonstrated acceptable treatment adherence before the trial phase. During the pilot phase and the trial, each therapist received weekly group supervision from two supervisors, as well as individual supervision. All supervisions made extensive use of videotaped sessions for feedback. The supervisors were licensed clinical psychologists, with extensive supervision experience. They received supervision concerning the supervision process from an international expert in SE, with more than 20 years of experience in psychodynamic treatment for depression, and more than 15 years of experience in SE treatments in RCTs. Two of the therapists did not continue after the training phase (one being offered a full-time position elsewhere, the other demonstrating low levels of adherence). Six therapists participated in the study. Their mean age was 42.33 (*SD*=4.41), and five were female. All were married or cohabitating. Their mean years of experience was 14.41 (*SD*=5.42). All therapists had psychodynamic training, two also had CBT training, and one also had biofeedback training. The mean number of patients each therapist treated was 16.66. (SD=8.45), range 5-31.
